# Sabine County Medical Society

**Published:** 1902-08

**Authors:** 


### Society Notes.

### Sabine County Medical Society.

The physicians of Sabine county met at Hemphill, Texas, in December last and organized the Sabine County Medical Society. Dr. J. W. Smith, of Fairmount, was elected President, and Dr. W. T. Arnold, of Hemphill, Secretary. The society held a very interesting meeting in July; they meet every month. Thus the good work of organization goes on. Now, if the members will all enroll under the banner of the "Red-Back" they can keep up with the procession, and will have music all the way there and back.

